# Principal variable selection to explain grain yield variation in winter wheat from features extracted from UAV imagery

**DOI:** 10.1186/s13007-019-0508-7

**Published:** 2019-11-01

**Authors:** Jiating Li, Arun-Narenthiran Veeranampalayam-Sivakumar, Madhav Bhatta, Nicholas D. Garst, Hannah Stoll, P. Stephen Baenziger, Vikas Belamkar, Reka Howard, Yufeng Ge, Yeyin Shi

**Affiliations:** 1 Department of Biological Systems Engineering, University of Nebraska-Lincoln, Lincoln, NE 68583 USA; 2 Department of Agronomy, University of Wisconsin-Madison, Madison, WI 53706 USA; 3 Department of Agronomy and Horticulture, University of Nebraska-Lincoln, Lincoln, NE 68583 USA; 4 Department of Statistics, University of Nebraska-Lincoln, Lincoln, NE 68583 USA

**Keywords:** Unmanned aerial vehicle, Phenotyping, Yield prediction, LASSO, Random forest, Ridge regression, SVM

## Abstract

**Background:**

Automated phenotyping technologies are continually advancing the breeding process. However, collecting various secondary traits throughout the growing season and processing massive amounts of data still take great efforts and time. Selecting a minimum number of secondary traits that have the maximum predictive power has the potential to reduce phenotyping efforts. The objective of this study was to select principal features extracted from UAV imagery and critical growth stages that contributed the most in explaining winter wheat grain yield. Five dates of multispectral images and seven dates of RGB images were collected by a UAV system during the spring growing season in 2018. Two classes of features (variables), totaling to 172 variables, were extracted for each plot from the vegetation index and plant height maps, including pixel statistics and dynamic growth rates. A parametric algorithm, LASSO regression (the least angle and shrinkage selection operator), and a non-parametric algorithm, random forest, were applied for variable selection. The regression coefficients estimated by LASSO and the permutation importance scores provided by random forest were used to determine the ten most important variables influencing grain yield from each algorithm.

**Results:**

Both selection algorithms assigned the highest importance score to the variables related with plant height around the grain filling stage. Some vegetation indices related variables were also selected by the algorithms mainly at earlier to mid growth stages and during the senescence. Compared with the yield prediction using all 172 variables derived from measured phenotypes, using the selected variables performed comparable or even better. We also noticed that the prediction accuracy on the adapted NE lines (*r* = 0.58–0.81) was higher than the other lines (*r* = 0.21–0.59) included in this study with different genetic backgrounds.

**Conclusions:**

With the ultra-high resolution plot imagery obtained by the UAS-based phenotyping we are now able to derive more features, such as the variation of plant height or vegetation indices within a plot other than just an averaged number, that are potentially very useful for the breeding purpose. However, too many features or variables can be derived in this way. The promising results from this study suggests that the selected set from those variables can have comparable prediction accuracies on the grain yield prediction than the full set of them but possibly resulting in a better allocation of efforts and resources on phenotypic data collection and processing.

## Background

As one of the leading sources for food production, wheat has the highest hectare over the world [1, 2]. Boosting grain yield to feed the ever growing world population is one of the major focuses in wheat breeding [3]. Recent developments in high-throughput field-based plant phenotyping have spawned various studies in wheat breeding programs, including using sensor-derived secondary traits to predict the primary trait of interest, such as yield. Accurate prediction of the primary trait can improve accuracy of genotypic selection, thus shortening breeding cycles and save costs. Two major sensing platforms have been widely used to measure the secondary traits in field: the ground-based and the aerial-based sensor platforms. Ground-based platforms provide large sensor payloads and throughputs. For example, a multi-sensor cart was developed for soybean and wheat breeding [4], mounted with ultrasonic sensor, NDVI sensor, thermal infrared radiometer, spectrometer, RGB sensor, as well as other ancillary sensors. Similar platforms include ‘phenocart’ [5], mobile ‘PhenoTrac’ [6, 7], and tractor-based semi-automatic system [8]. As for aerial-based platforms, the unmanned aerial vehicle (UAV) is gaining increased attention due to ease of operation, high spatial resolution, and quick coverage [9–12]. Typical sensors equipped on UAV in agricultural applications are RGB cameras [13], multispectral cameras [14], thermal cameras [15], and hyperspectral sensors [16].

It is of a great interest to use UAV-derived phenotypic traits for yield prediction. For winter wheat, the grain yield is usually estimated by vegetation indices [14, 17, 18] or morphological traits derived from aerial imagery at single growth stage [19]. For example, by deriving normalized difference vegetation index (NDVI) from UAV imagery on different growth stages, the highest correlation coefficient (*r*) of 0.91 was found between NDVI and final yield around flowering time [14]. In addition to looking at single growth-stage, researchers also attempted to exploit extra predictive power by integrating phenotypic traits from multiple growth-stages. In the study of Du & Noguchi [13], five RGB indices accumulated over eight flights were used as variables in the stepwise regression model, and the best model with four indices was selected (*r *= 0.69 on validation set). Additionally, Haghighattalab et al. [10] input multi-temporal phenotypic traits into principal component regression (PCR) and geographically weighted (GW) model to estimate wheat yield. The GW model considered the spatial relationship among acquired images, which performed better on grain yield prediction than PCR (*r* increased from 0.26 to 0.74 under the drought environment, and from 0.24 to 0.46 under irrigated environment).

Despite the promising findings on yield prediction with remotely sensed phenotypic traits throughout the growing season [20, 21], collecting and processing multi-temporal traits is still time-consuming and computationally expensive. For example, in this study, data collection on the winter wheat during spring growing season started from mid-April to mid-June in 2018 on a weekly basis. The collected imagery data after each flight took approximately 30 gigabytes of storage (around 9000 multispectral images and 1000 RGB images, over the 1.2 hectare field). Currently, processing such large dataset in a short time is still complicated. If several key UAV-derived phenotypic traits or growth stages for grain yield are available, data collection and processing efforts could be streamlined. The predictive model will also be simplified, allowing a better understanding of the predictive power of individual traits.

To determine critical phenotypic traits or growth stages, variable selection algorithms can be performed to reserve principal predictors, which in this case are features extracted from UAV imagery, based on the predictive powers of individual predictors on the response variable, which can be grain yield for example [22]. In this way, further processing can be narrowed down to those selected principal variables with reduced computational complexity, improved data analysis efficiency, and better data understanding [23, 24]. In this study, we adopted two common variable selection algorithms: LASSO regression and random forest. LASSO was firstly proposed by Tibshirani [25]. It adds penalty into parameter estimation to shrink the near-zero regression coefficients to zero, thus removing them out of the selection result. Random forest [26] aggregates hundreds of individual decision trees to achieve a better trade-off between bias and variance [27, 28]. It is a ranking-based nonparametric selection algorithm [28, 29], providing importance measurement for each variable. Both LASSO and random forest are feasible when the number of variables is greater than the number of observations [30, 31].

Only a few studies investigated the principal variable selection on UAV-derived phenotypic traits for wheat grain yield prediction [10, 13]. Furthermore, more features could be extracted from the ultra-high spatial resolution UAV images rather than common averaged statistical descriptions at the plot level (e.g., mean vegetation indices of each plot) [32–38]. In addition, it is meaningful to examine the predictive power of dynamic features from the multi-temporal UAV data, such as growth rate. To this end, the objective of this study was to select principal phenotypic variables that contribute most in explaining the grain yield in winter wheat, to potentially reduce the efforts in field phenotyping data collection and the subsequent data processing. Two specific objectives were:

1. To maximize the feature/variable extractions from the UAV-derived vegetation index (VI) and plant height maps including pixel statistics (e.g., mean, median) and dynamic growth rate.
2. To perform principal variable selection on extracted variables, and to evaluate predictive power for grain yield using the selected principal variables.

Clarification of terminology in this study:

- Primary trait: grain yield;
- Secondary traits: plant height, spectral reflectance;
- UAV derived maps: plant height, NDVI, NDRE, and GNDVI map;
- Features (variables) extracted from individual plots in UAV derived maps: trimmed mean, median, mode, 95th percentile, standard deviation, contrast, correlation, energy, and homogeneity.

## Methods

### Field layout

**Fig. 1:**
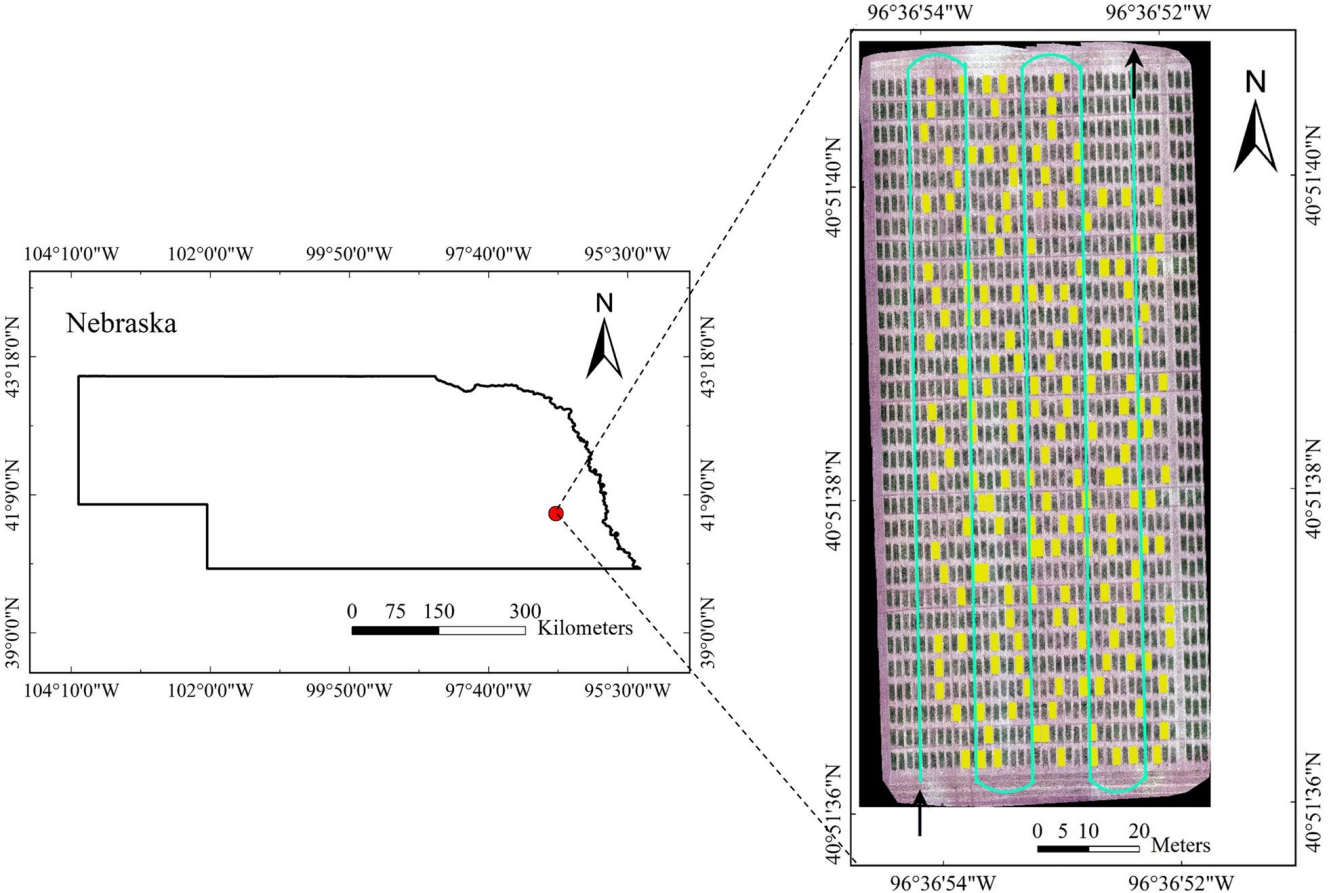
Field location and layout. The field was located in eastern Nebraska, USA (left). Cyan line indicates the flight path, and yellow rectangles indicate the studied 170 plots (right). The map was generated using images collected on April 27, 2018

The studied field was located in Lincoln, Nebraska, USA (N 40.8581, W 96.6157), where winter wheat was grown during the growing season from the end of October, 2017 to early July, 2018. As part of a larger augmented design for yield trial, ten check lines with 17 replications, in total 170 plots, were used in this study (Fig. 1). The ten checks include three Nebraska (NE) lines (Freeman, Robidoux, Ruth), three Texas (TX) lines (TAM 304, TAM 113, TAM 114), two Westbred (WB) lines (WB Cedar, WB Grainfield), one Oklahoma line (Gallagher), and one Syngenta line (SY Wolf). The remaining plots in this trial were reserved proprietary lines at the time of this study. The checks were grown in plots of five rows of 3.0 m length and with 0.23 m spacing between the rows. Each check plot was planted with 35 grams of seeds, with a seeding rate of approximately 1,000,000 seeds per acre. Grain yield was measured in all five rows of each plot in early July, using a Zurn 150 Combine harvester (Zurn, Schöntal-Westernhausen, Germany) with a weigh system on the combine [39].

### UAV system and flight missions

**Fig. 2:**
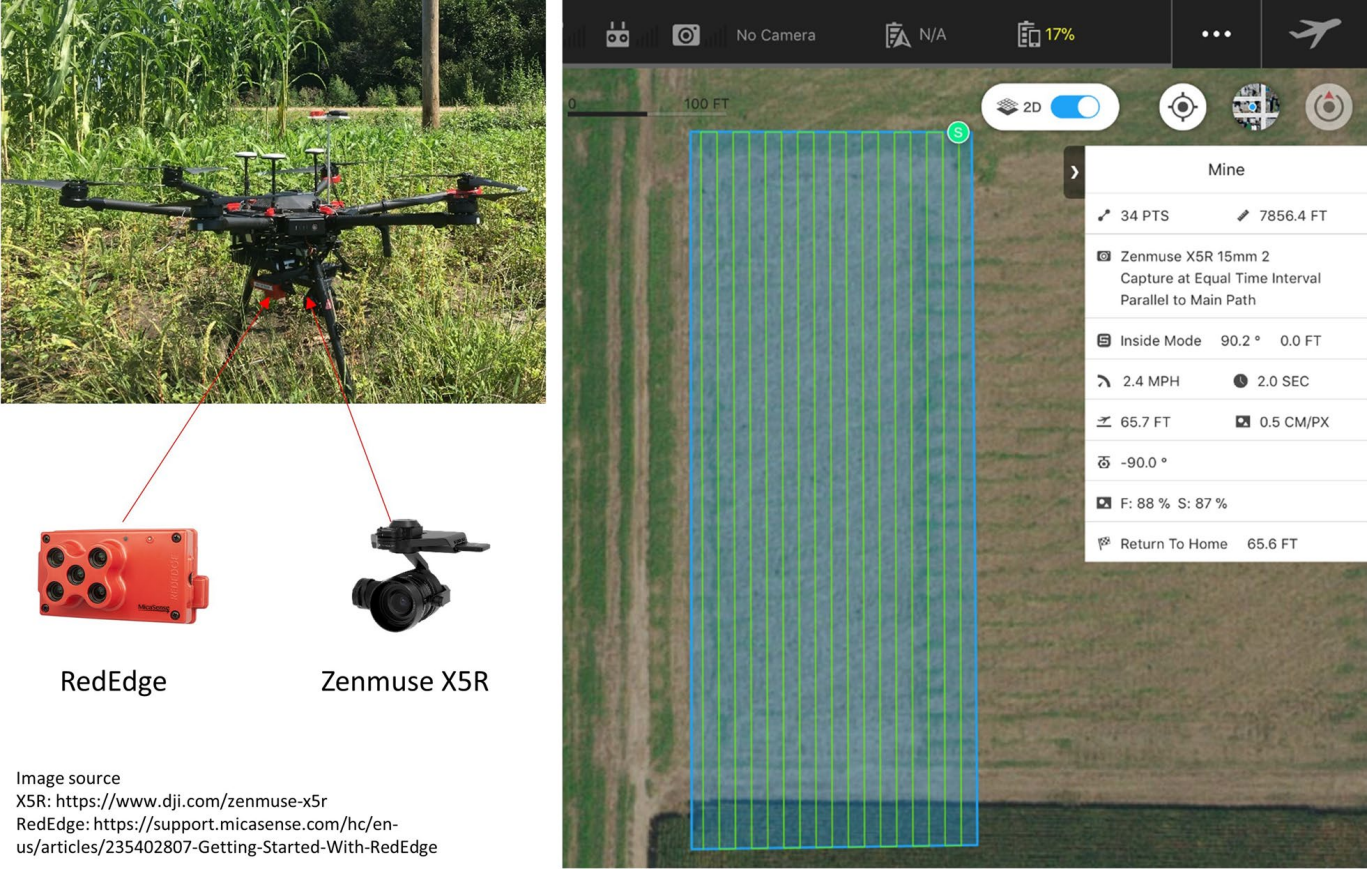
The UAV system (left) and the flight parameter settings on the DJI GS Pro application (right)

The UAV system used in this study consisted of a DJI Matrice 600 Pro multi-rotor platform (DJI, Shenzhen, China), a Zenmuse X5R RGB camera (DJI, Shenzhen, China), and a five-band multispectral camera RedEdge (Micasense, Seattle, USA) (Fig. 2). Each RGB image has an effective pixel size of 4608 by 3456, and each multispectral image has an effective pixel size of 1280 by 960. The multispectral camera also comes with a standard calibration panel for radiometric calibration, which was imaged on the ground right before or after each flight.

Seven RGB image sets and five multispectral image sets were acquired from mid-April to mid-June in 2018, and the corresponding growth stages are listed in Table 1. After several test flights, the flight altitude was set as 20 meters (65.7 FT in Fig. 2) above ground, and the forward and sideward overlaps were set as 88% and 87%, respectively. These flight parameters were set on the DJI GS pro application, as showed in Fig. 2 (right). The corresponding ground sampling distance (GSD) was 0.5 cm/pixel for the RGB image and 1.35 cm/pixel for the multispectral image. In order to do the geometric calibration, 21 ground control points (GCPs) using black and white cross-centered wooden boards were evenly placed over the field. GPS information of these GCPs was measured by a survey grade GNSS RTK GPS receiver (Topcon Positioning Systems, Inc., Tokyo, Japan), with centimeter accuracy in the X and Y directions, and centimeter accuracy in the Z direction. Three sources of data were used for the radiometric calibration: the data collected on the ground right before and after each flight over a MicaSense’s Calibrated Reflectance Panel, and the ambient light conditions in each of the five bands in a real time throughout the flight using a MicaSense Downwelling Light Sensor mounted on top of the UAV facing up towards the sky. The reflectance of the calibrated panel was 0.57, 0.57, 0.57, 0.56, and 0.53 in the blue, green, red, red edge, and near-infrared bands, respectively. In addition, standard calibration tarps were set up during data collections in the field with reflectance in 0.03, 0.22, and 0.48, respectively, to provide another source of information for radiometric calibration.

**Table 1:** Seven data collections over the spring season of 2018

Date	Acquired image type	Day of year (DOY)	Growth stage
April 22	RGB	111	Tillering stage: Feekes 3
April 27	RGB and Multispectral	116	Green-up stage: Feekes 5
May 7	RGB and Multispectral	126	Jointing stage: Feekes 6
May 15	RGB	134	Flag leaf stage: Feekes 8
May 21	RGB and Multispectral	140	Boot stage: Feekes 9
June 1	RGB and Multispectral	151	Grain filling: Feekes 10.5.3
June 18	RGB and Multispectral	168	Physiological maturity: Feekes 11

### Orthomosaicking and geometric and radiometric calibrations

Raw multispectral and RGB images were mosaicked or stitched using Pix4Dmapper (Pix4D, Lausanne, Switzerland), to further generate vegetation index (VI) and plant height maps [40]. Geometric calibration was performed during the orthomosaicking process in Pix4Dmapper using the GCPs setup at the data collection. Radiometric calibration included two steps. First one was the automatic radiometric calibration performed by Pix4Dmapper during the orthomosaicking process using the calibration data collected over the calibrated panel along with the ambient light changes collected during the flight from the downwelling light sensor. The output five-band maps from Pix4D were 16-bit GeoTIFFs, with pixel digital number ranging from 0 to 65,535. Pixel digital number in each map was further calibrated and converted to reflectance ratios ranging from 0 to 1, using the standard calibration tarps.

### Vegetation index and plant height maps generation

Targeted plots were delineated with equal size and specific ID in ArcMap for following information extractions (Fig. 1). The plant height maps were calculated as the difference between the digital surface model (DSM) and the digital terrain model (DTM). The DSM was created automatically in Pix4Dmapper, representing the elevation of the canopy surface. A DTM map represents the elevation of the soil surface. In this study, the DTM was created by interpolating segmented soil pixels. Specifically, the RGB map from the earliest flight that had the highest proportion of bare soil exposure was transferred into the CIELAB color space. The histogram of the A channel in this color space is generally considered as a Gaussian-mixture model of vegetation pixels and soil pixels [41], thus being useful in segmenting soil and vegetation pixels. According to the threshold calculation method described in Liu et al. [42], a mask with only soil pixels was created. From this mask, thousands of soil points were randomly sampled to create DTM using Kriging interpolation in ArcMap 10.5.1 (Esri Inc. CA, USA).

Three classical VIs were calculated from the 5-band multispectral maps in RStudio 1.0.153 (RStudio, Inc. Boston, USA): NDVI, green NDVI (GNDVI), and normalized difference red edge (NDRE) (Eqs. 1–3). These traits are highly correlated with leaf chlorophyll contents and canopy structures, therefore, they are widely used for yield predictions [12, 14, 17, 18].

1 $$NDVI = \left( {R_{NIR} - R_{Red} } \right)/\left( {R_{NIR} + R_{Red} } \right)$$

2 $$GNDVI = \left( {R_{NIR} - R_{Green} } \right)/\left( {R_{NIR} + R_{Green} } \right)$$

3 $$NDRE = \left( {R_{NIR} - R_{Red - edge} } \right)/\left( {R_{NIR} + R_{Red - edge} } \right)$$

where *R* stands for the reflectance of the spectral band indicated in the subscript.

### Variables extracted from VI and plant height maps

Two classes of variables were extracted in this study: pixel statistics and dynamic growth rate. For the pixel statistics, in addition to commonly extracted statistics (e.g., mean or median), each plot was further transferred into a texture feature matrix, i.e., gray-level co-occurrence matrix (GLCM), to derive statistical texture variables (contrast, correlation, energy, and homogeneity). The GLCM is a feature extraction method, allowing the extraction of second-order statistical texture variables [43]. The ‘second-order’ means that GLCM only considers the relationship between two pixels. Specifically, from un-transferred VI map, trimmed mean (mean value after trimming top and bottom 10% values), median (equals to 50th percentile), mode, and standard deviation were derived for each plot; similarly from un-transferred plant height map, trimmed mean, median, 95th percentile, and standard deviation were derived for each plot. After transferring each map into GLCM, another four statistical texture variables were calculated: contrast, correlation, energy, and homogeneity. Contrast represents the local gray level variations in an image; high contrast indicates the existence of any edges, noise, or wrinkled texture. Correlation measures the linear dependency of specified pixel pairs. Energy, also known as angular second moment, sums up the squared elements in GLCM. As for homogeneity, it is also called inverse difference moment and stands for the local homogeneity; high value represents the uniform local gray level.

**Fig. 3:**
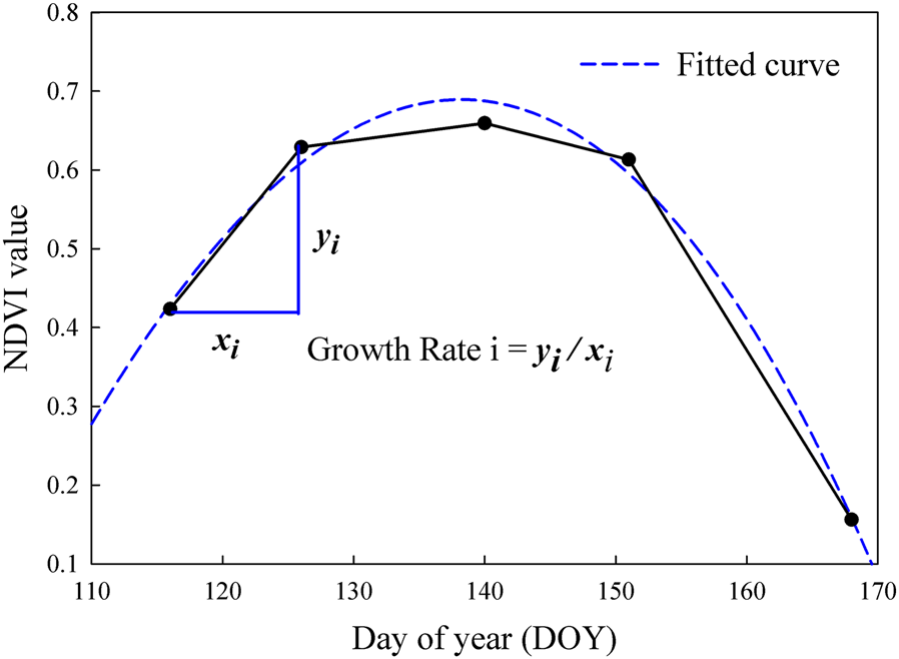
Dynamic growth rate calculation based on the dynamic curve of NDVI

The second class of variable was dynamic growth rate, defined as the slope between two successive measurements along the season. In this study, dynamic growth rates were calculated for individual plots using the trimmed means of NDVI, NDRE, GNDVI, and plant height, respectively. An example was illustrated using NDVI dynamic curve in Fig. 3. With five NDVI measurements along the season, four growth rates were calculated with a negative number for the last one. The negative growth rate indicates the senescence process. As for plant height, with seven time-points in the dynamic curve (Fig. 5b), six growth rates were calculated.

Among the first class of variables, a significantly strong correlation (*r* = 0.99) was found between the trimmed mean and the median value. Therefore, the trimmed mean value was only used to calculate the dynamic growth rate and was not input into variable selection algorithms. Summing up two classes of variables, there were 172 variables for each experimental plot, as summarized in Table 2.

**Table 2:** Summary of the 172 variables in each plot extracted from VI and plant height maps

UAV derived map	Number of variables	
	Pixel statistics	Dynamic growth rate
Plant height	7 features × 7 dates = 49*	6
NDVI	7 features × 5 dates = 35**	4
NDRE	7 features × 5 dates = 35**	4
GNDVI	7 features × 5 dates = 35**	4
Total number of variables	172	

* Median, 95th percentile, standard deviation, contrast, correlation, energy, and homogeneity

** Median, mode, standard deviation, contrast, correlation, energy, and homogeneity

### Principal variable selection for grain yield estimation

The extracted 172 variables were treated as candidates in two variable selection algorithms to explain grain yield. Normalization was conducted before each selection procedure. The main parameter tuned in LASSO was lambda, a shrinkage penalty term. It was tuned through 10-fold cross-validation, with mean squared error (MSE) as the loss function. Lambda shrank some variable coefficients to zero while retaining non-zero variables. In addition to variable selection, LASSO also estimates the regression coefficients for selected variables. Since all variables were normalized, variables with higher absolute coefficient could be considered as contributing more in explaining grain yield. Therefore, the absolute regression coefficient was used as the ‘importance score’ for the variable selected by LASSO.

Random forest ranks variables according to the permutation importance of each variable. Generally, if a variable X is important for the dependent variable Y, permuting the order of X will break the correlation link between X and Y, thus increasing prediction error (MSE) [27]. In other words, the higher the increase in MSE after permuting variable X, the more critical the variable X is. Therefore, the increase in MSE (%IncMSE) was chose as the ‘importance score’ in random forest selection. Parameter tuned for the random forest in this study was the number of trees to grow, and the number of variables randomly sampled as candidates at each split. Both were optimized by grid search and were set as 1500 and 2 separately.

Compared to random forest, LASSO is more sensitive to multicollinearity among variables [44]. When there is a group of correlated variables, LASSO would arbitrarily select one variable from this group at each random run, thus resulting in inconsistent selections [45]. To alleviate this effect, each algorithm was set to run 30 times with different random seeds. Afterward, each variable had two lists of importance scores, with a length of 30, from LASSO and random forest. Ten variables with the highest averaged importance scores were chosen for LASSO and random forest, respectively.

To further evaluate the ability of grain yield prediction using the selected variable sets versus the original variables, ridge regression and Gaussian kernel-based support vector machine (SVM, non-parametric) were applied. Ridge regression is a parametric prediction algorithm. It is capable of addressing the collinearity issue that was not handled by multiple linear regression [46–48]. SVM can be used to solve classification and regression problems by constructing a hyperplane with maximized margins for separation in a high-dimensional space. The use of Gaussian radial basis function allows the SVM model to address nonlinearity data [49]. For each model, 80% observations were used as training data and 20% observations were treated as testing data. The predicting performance of each model using the selected variable sets was compared with the performance using all 172 variables and the correlation coefficients (*r*) and the root mean squared errors (*RMSE*) were shown. In addition, performance on lines grouped by their genetic background were also investigated, i.e., the NE lines, TX lines, WB lines, and two other lines (OK and SY line).

## Results and discussion

### Growth dynamics in terms of VI and plant height

**Fig. 4:**
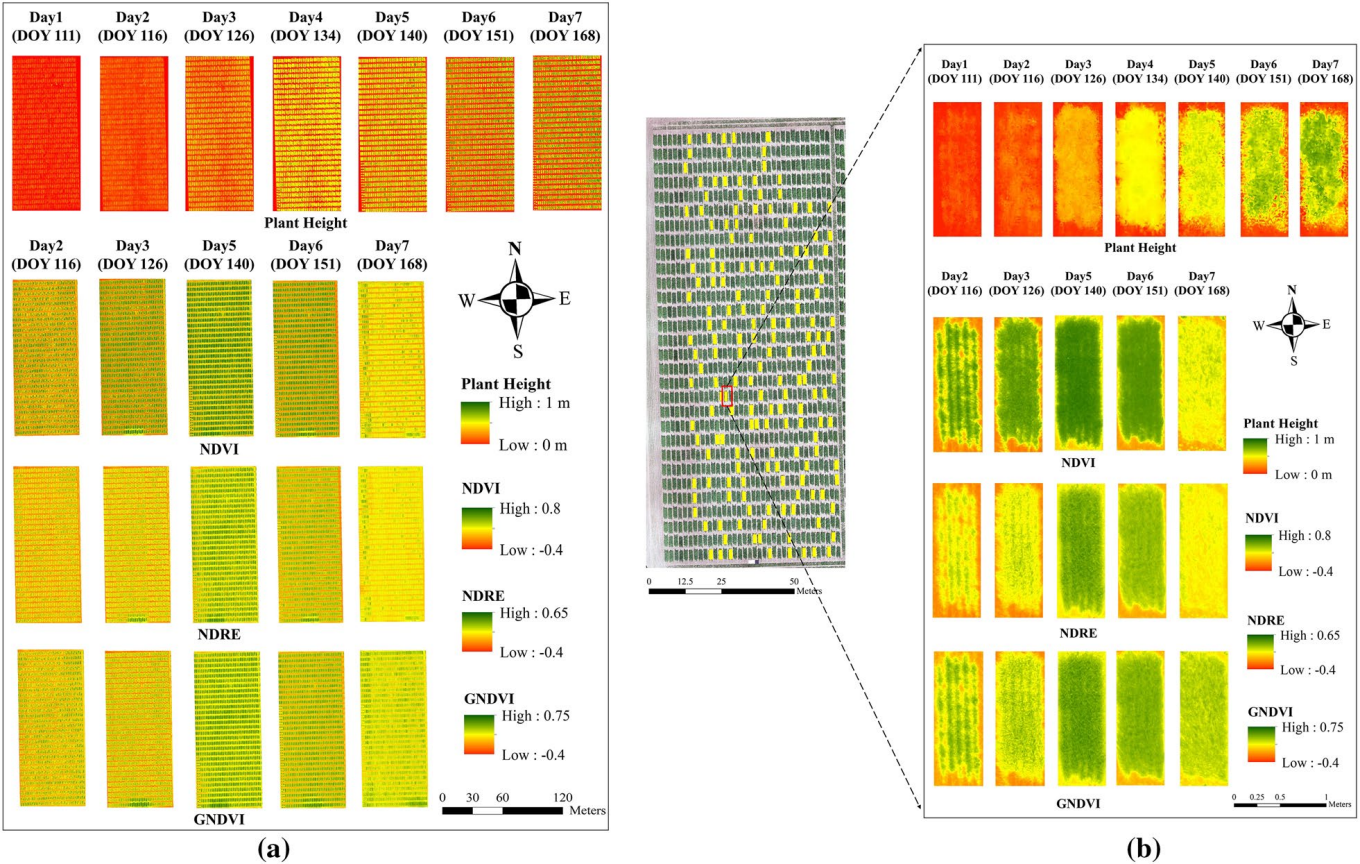
Multi-temporal maps of plant height and three vegetation indices. DOY represents ‘day of year’ for each date, corresponding to x-axis in Fig. 5. **a** Whole field maps. **b** An example plot selected randomly (field map used images collected on May 7, 2019)

One application of the multi-temporal UAV data was to track the growth trend of winter wheat, using UAV-derived maps and dynamic curves. The multi-temporal maps tracking seasonal growth of the whole field was provided in Fig. 4a. Additionally, to visualize the growth trend of specific plot, one plot was randomly selected as the example and was presented in terms of plant height and VIs (Fig. 4b). Greener pixel of plant height means taller wheat plant, while greener pixel of VI indicates greater wheat vigor. As expected, plant height increased over the spring growing season; whereas the VI value peaked and had the greatest vigor on the 140 DOY. Among three VIs, a significant saturation issue could be found in NDVI around 140 DOY, which agrees with most other studies [50, 51] that NDVI tends to saturate with dense canopy cover.

**Fig. 5:**
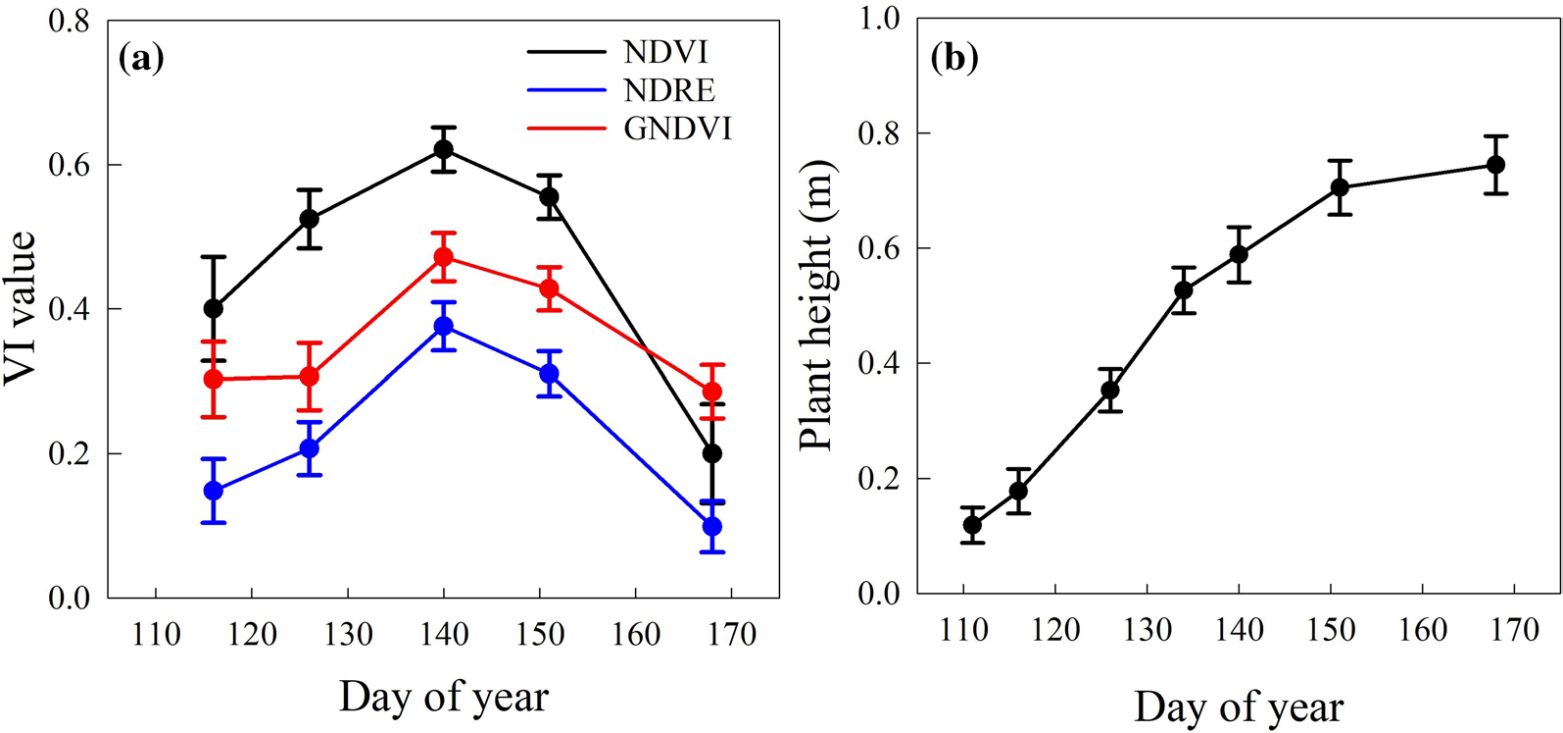
Growth dynamic curves of VIs (**a**) and plant height (**b**) over the spring growing season in 2018. Error bars represent standard deviation among the 170 plots

The dynamic curves provide a quantitative way to describe growth trend, using a trimmed mean of plant height or VI (Fig. 5). The NDVI, GNDVI, and NDRE followed a similar growth trend that reached a peak at the 140 DOY, with a significant drop after the 151 DOY (Fig. 5a). The dynamic curve also exhibited different growth rates over the season. For example, NDVI had almost equal growth rates between the 116 and 140 DOY, whereas NDRE and GNDVI had a smaller growth rate between the 116 and 126 DOY than that between the 126 and 140 DOY. In Fig. 5b, the plant height dynamic curve showed an increasing trend along seven data collections.

Both maps and dynamic curves exhibited similar winter wheat growth trends (Figs. 4 and 5): VI reached peak value around May 21 (boot stage) and plant height increased until June 18 (physiological maturity). The significantly low VI values on the last data collection were due to leaf yellowing at the end of the growth season. The seasonal changes of these VIs were similar to previous research [8, 52]. Similarly, the growth trend of plant height was also found in related research [36, 53], followed the pattern of the sigmoid curve. It was also noticeable that VI curves started to drop around May 21, whereas the growth rate of plant height decreased significantly after June 1. Considering the manually recorded flowering date (from end-May to early-June), it is possible that these changes, i.e., VI vigor starting to drop and height growth rate beginning to decrease, were synchronized with flowering.

### Variable selection by LASSO and random forest

**Fig. 6:**
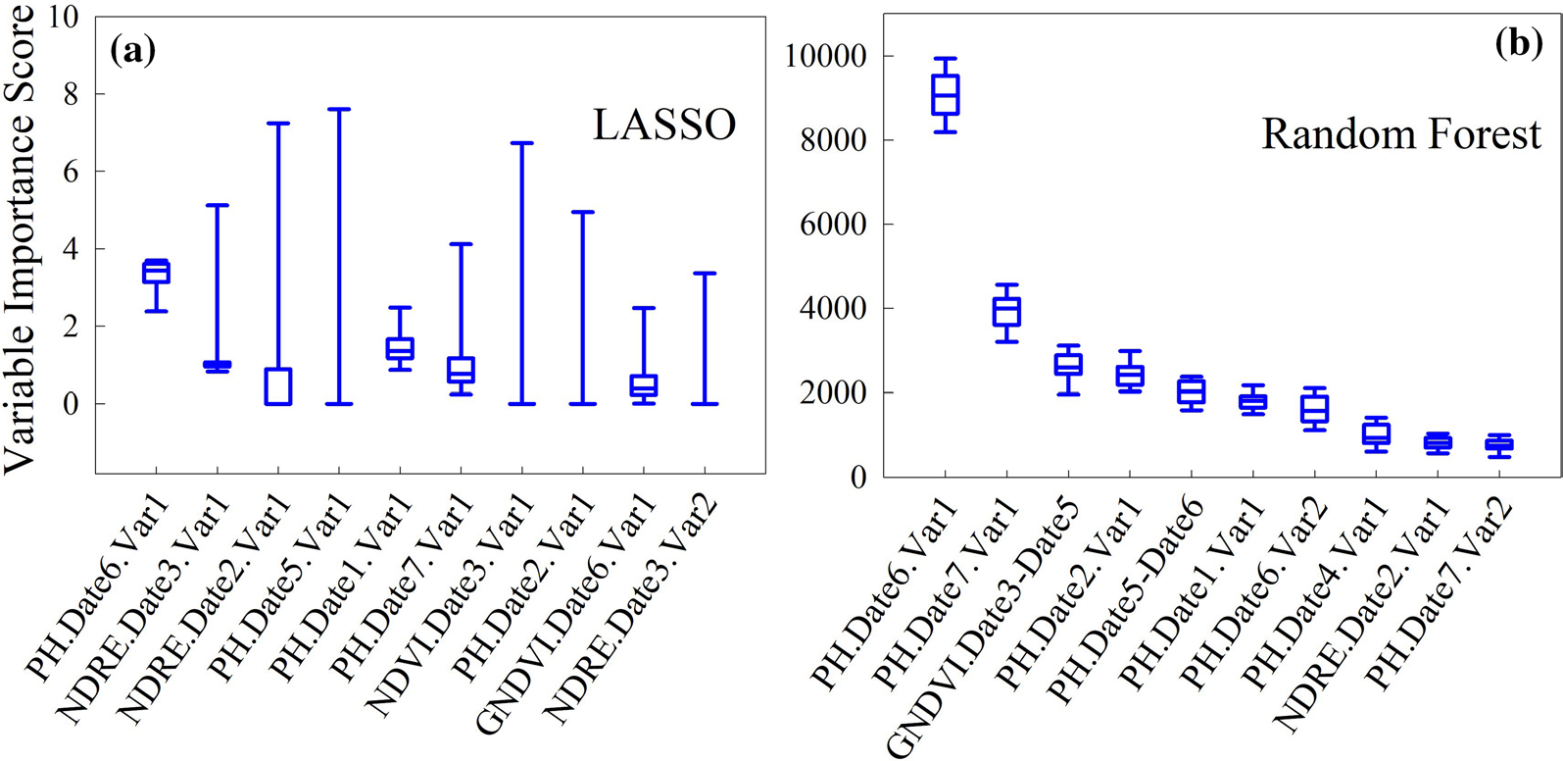
Variable importance scores of top 10 variables selected by LASSO and random forest

LASSO and random forest selected top ten variables were ranked in Table 3, according to averaged importance score. Each row represents one variable, with details of specific variable name, the corresponded map and on which times of data collection that the variable was derived from. Besides, an abbreviation was given for each selected variable and shown in x-axis of Fig. 6, with the purpose of presenting summarized variable importance scores. Two common variables selected by both algorithms were: PH.Date6.Var1 and NDRE.Date2.Var1.

**Table 3:** Ten variables selected by LASSO and random forest, respectively

Rank	Feature	Originated map	Date	Abbreviation
LASSO selected variables				
1	Median	Plant height	6th	*PH.Date6.Var1*
2	Contrast	NDRE	3rd	NDRE.Date3.Var1
3	Standard deviation	NDRE	2nd	*NDRE.Date2.Var1*
4	95th percentile	Plant height	5th	PH.Date5.Var1
5	95th percentile	Plant height	1st	PH.Date1.Var1
6	Homogeneity	Plant height	7th	PH.Date7.Var1
7	Standard deviation	NDVI	3rd	NDVI.Date3.Var1
8	Standard deviation	Plant height	2nd	PH.Date2.Var1
9	Correlation	GNDVI	6th	GNDVI.Date6.Var1
10	Standard deviation	NDRE	3rd	NDRE.Date3.Var2
Random forest selected variables				
1	Median	Plant height	6th	*PH.Date6.Var1*
2	Median	Plant height	7th	PH.Date7.Var1
3	Second growth rate	GNDVI	3rd and 5th	GNDVI. Date3-Date5
4	95th percentile	Plant height	2nd	PH.Date2.Var1
5	Fifth growth rate	Plant height	5th and 6th	PH.Date5-Date6
6	Correlation	Plant height	1st	PH.Date1.Var1
7	95th percentile	Plant height	6th	PH.Date6.Var2
8	Median	Plant height	4th	PH.Date4.Var1
9	Standard deviation	NDRE	2nd	*NDRE.Date2.Var1*
10	95th percentile	Plant height	7th	PH.Date7.Var2

Common variables selected by both algorithms were given in italic

PH.Date6.Var1, corresponded to the median value of the canopy height measurements within a plot around the grain filling stage (Table 3), was ranked as the top one by both models. The grain filling stage has already been proved to be critical for assessing wheat grain yield in previous research [20, 54, 55]. Both models selected more plant height related variables over VI related ones in this case. Though results may change in another case, this still confirmed the importance of plant height in wheat grain yield prediction. In fact, the relationship between plant height and wheat yield has always been an interesting topic for breeders. Back in 1978, Law [56] found a positive relationship between plant height and wheat yield. Further, with wheat plant height measured over multiple growth stages, strong positive correlations were found between plant height and final grain yield [57]. What is new with the UAS-based phenotyping compared with the traditional methods is that we are now able to obtain ultra-high resolution plot imagery to derive more features, such as the variation of plant height or vegetation indices within a plot other than just an averaged number, that are potentially very useful in breeding.

The potential importance of plant height, however, does not necessary mean that the vegetation indices are not useful in explaining grain yield. Good performance of VI on grain yield prediction have already be demonstrated in many studies [58, 59]. NDRE.Date2.Var1, another common variable selected by LASSO and random forest, was ranked as top third variable by LASSO. However, different from PH.Date6.Var1 that was derived at later growth stage, NDRE.Date2.Var1 was calculated from earlier growth stages. Besides, most of the other VI related variables selected were derived on earlier dates. This finding potentially indicates the importance of VI variables derived at earlier growth stages. In addition, compare to the most commonly known NDVI, NDRE and GNDVI related variables seem to appear more frequently in the selection results. As mentioned above, NDVI tends to saturate earlier than NDRE and GNDVI, which possibly result in less NDVI variables being selected.

Figure 6 summarized the importance scores of selected variables from 30 random runs. It was observed that the selection results of random forest were more consistent than those of LASSO. As mentioned above, since LASSO has higher sensitivity to multicollinearity among variables than random forest [44], the inconsistent results of LASSO indicated a possible multicollinearity issue among variables. Running the algorithm for 30 times randomly was the method adopted in this study to alleviate influence from this issue. Another solution that could be considered in future work is, to cluster correlated variables into groups first and then do selection on representative variables from each group [60, 61].

### Grain yield prediction using selected variable sets

With the two sets of selected principal variables, the grain yield was estimated using both ridge regression and SVM model with Gaussian radial basis kernel. Performances on testing data (20%) were reported in Table 4, with *r* and RMSE averaged from 20 random sets of testing data.

**Table 4:** Performance of grain yield prediction on testing data, using variable sets determined from LASSO and random forest, as well as all available variables

Variables	LASSO selected variables		Random forest selected variables		All 172 variables	
Sample size	r*	RMSE* (g/plot)	*r*	RMSE (g/plot)	*r*	RMSE (g/plot)
(1) Predictions of SVM model with Gaussian radial basis kernel						
All lines	0.32	320.19	0.39	306.15	0.29	314.77
NE lines	0.58	326.97	0.77	254.66	0.72	284.08
TX lines	0.21	271.44	0.36	255.51	0.57	215.92
WB lines	0.28	271.88	0.41	236.82	0.25	264.53
OK and SY lines	0.39	201.45	0.45	191.06	0.36	193.31
(2) Predictions of ridge regression model						
All lines	0.49	283.86	0.39	301.89	0.25	314.83
NE lines	0.73	272.72	0.81	225.45	0.73	295.92
TX lines	0.55	235.37	0.50	255.68	0.47	242.99
WB lines	0.40	247.57	0.42	246.21	0.22	266.25
OK and SY lines	0.59	163.69	0.54	164.90	0.58	169.29

* Values of *r* and RMSE were averaged from 20 random sets of testing data

Comparing among three different variable sets, random forest selected variable set with SVM model had a slightly higher prediction accuracies (*r* = 0.36–0.77) than the other two variable sets (*r* = 0.21–0.58 for the LASSO selected set, and *r* = 0.25–0.72 for all variables); whereas the LASSO selected variable set with ridge regression (*r* = 0.40–0.73) had relatively but not significantly better performance than the other two sets (*r* = 0.39–0.81 in random forest selected set, and *r* = 0.22–0.73 in all variables set). It is noticeable that both random forest and SVM are non-parametric algorithm, while both LASSO and ridge regression are parametric algorithm. A possible suggestion could be made is that, non-parametric prediction model could be adopted to match the non-parametric variable selection, and vice versa. Another finding by comparing the three variable sets is that, the overall performance of using two selected variable sets with greatly reduced number of variables was better than using whole 172 variables.

**Fig. 7:**
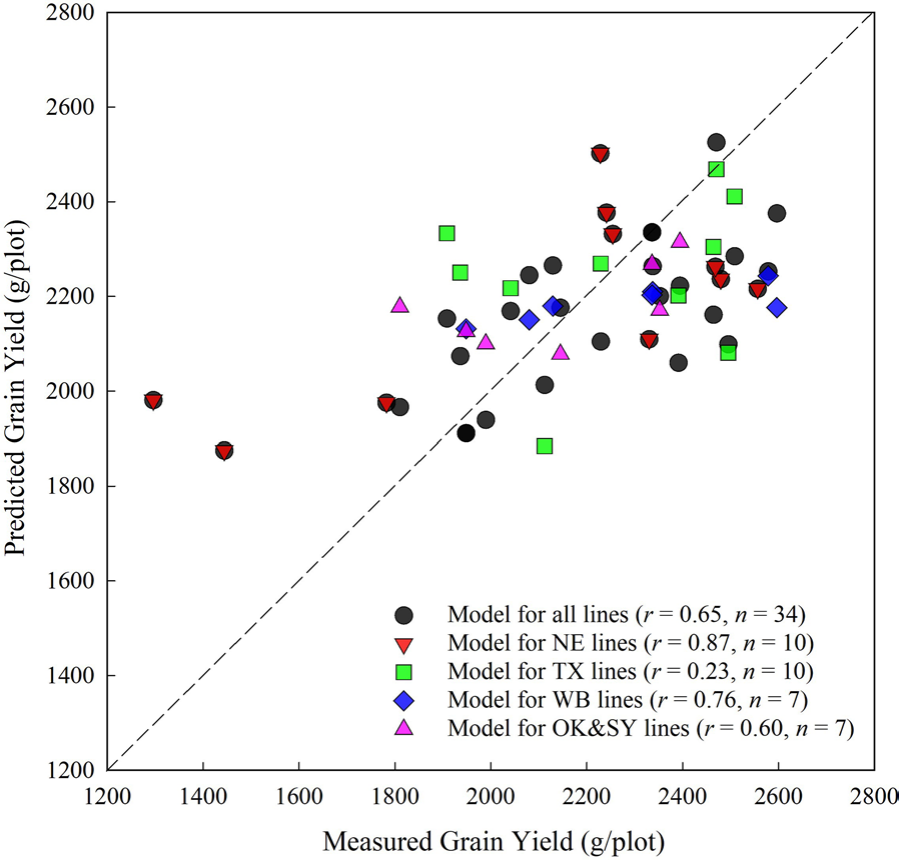
Relationship between measured and predicted grain yield of an example training–testing set for each group of lines. Dashed line is the 1:1 line. *r* is correlation coefficient; *n* is testing sample size

The selected variables actually performed better on individual grouped lines with different genetic background than pooling all ten lines together (Table 4). The highest prediction performance was achieved for the NE lines (*r* = 0.58–0.81). Except for the NE lines which were bred for NE environments, the rest of them were bred for their target environments. Use the WB Cedar line as an example. This is a relatively shorter line and flowers at least a week before the NE lines. Hence, the variables that were picked as being most important might be different for Cedar compared to NE lines. And the way that we used sampling dates as opposed to developmental stages in the study would also exaggerate the issue. The observations with all 10 checks could be confounded by the different genetic backgrounds and development patterns. When analyzed them separately, this confounding effect was avoided and that is probably why the results looked a lot better. Even for a single line, there existed much variation in the field. For example, the yield of Freeman ranges from 1057 g (35.2 bu/a) to 1813 g (60.4 bu/a)—variation of nearly 25 bushels per acre. The variation can be expected to be even larger among those un-adapted lines. To illustrate the information in Table 4, the predicted grain yield of a random training–testing set with the ridge regression model was plotted versus measured yield (Fig. 7). The results of pooled ten lines and individual group of lines were shown.

## Conclusions

This study investigated the principal variable selection from features extracted from UAV-derived imagery for winter wheat grain yield prediction using LASSO and random forest algorithms. Selection results showed that plant height related variable derived at grain filling stage was ranked as the top one by both LASSO and random forest models; whereas temporal plant height and VIs are important throughout the season. Furthermore, the yield prediction using reduced variable sets selected by LASSO or random forest had comparable or even better accuracy than using all extracted variables. This indicated the possibility of a better allocation of efforts and resources on phenotypic data collection and processing by narrowing down the targeted secondary traits and growth stages. What can be noticed is that with the ultra-high resolution plot imagery obtained by the UAS-based phenotyping we are now able to derive more features, such as the variation of plant height or vegetation indices within a plot other than just an averaged number, that are potentially very useful for the breeding purpose. Further studies can be conducted to investigate the potential of genomic prediction by incorporating the selected key secondary traits measured by sensing systems into the genomic prediction models to increase the prediction accuracy. This study also serves as a preliminary study for future experiments, where the proposed variable selection methodology can be tested with more data collected in multiple years and locations to streamline the phenotyping to support the breeding process.

## Data Availability

The datasets used and/or analyzed during the current study are available from the corresponding author on reasonable request.
